# Selinexor, bortezomib, and dexamethasone versus bortezomib and dexamethasone in previously treated multiple myeloma: Outcomes by cytogenetic risk

**DOI:** 10.1002/ajh.26261

**Published:** 2021-07-05

**Authors:** Shambavi Richard, Ajai Chari, Sosana Delimpasi, Maryana Simonova, Ivan Spicka, Ludek Pour, Iryna Kriachok, Meletios A. Dimopoulos, Halyna Pylypenko, Holger W. Auner, Xavier Leleu, Ganna Usenko, Roman Hajek, Reuben Benjamin, Tuphan Kanti Dolai, Dinesh Kumar Sinha, Christopher P. Venner, Mamta Garg, Don Ambrose Stevens, Hang Quach, Sundar Jagannath, Phillipe Moreau, Moshe Levy, Ashraf Badros, Larry D. Anderson, Nizar J. Bahlis, Thierry Facon, Maria Victoria Mateos, Michele Cavo, Hua Chang, Yosef Landesman, Yi Chai, Melina Arazy, Jatin Shah, Sharon Shacham, Michael G. Kauffman, Sebastian Grosicki, Paul G. Richardson

**Affiliations:** ^1^ Icahn School of Medicine at Mount Sinai Tisch Cancer Institute New York New York USA; ^2^ General Hospital Evangelismos Athens Greece; ^3^ Institute of Blood Pathology & Transfusion Medicine of National Academy of Medical Sciences of Ukraine Lviv Ukraine; ^4^ Charles University and General Hospital Prague Czech Republic; ^5^ Clinic of Internal Medicine —Hematology and Oncology University Hospital Brno Brno Czech Republic; ^6^ National Cancer Institute Ukraine Kiev Ukraine; ^7^ School of Medicine National and Kapodistrian University of Athens School of Medicine Athens Greece; ^8^ Department of Hematology Cherkassy Regional Oncological Center Cherkassy Ukraine; ^9^ Imperial College London London UK; ^10^ Department of Hematology CHU la Miletrie and Inserm CIC 1402 Poitiers France; ^11^ City Clinical Hospital No. 4 of Dnipro City Council Dnipro Ukraine; ^12^ Department of Hemato‐oncology, University Hospital Ostrava University of Ostrava Ostrava Czech Republic; ^13^ Kings College Hospital NHS Foundation Trust London UK; ^14^ Nil Ratan Sircar Medical College and Hospital Kolkata India; ^15^ State Cancer Institute Indira Gandhi Institute of Medical Sciences Patna India; ^16^ Cross Cancer Institute University of Alberta Edmonton Alberta Canada; ^17^ University Hospitals of Leicester NHS Trust Leicester UK; ^18^ Norton Cancer Institute St. Matthews Campus Louisville Kentucky USA; ^19^ University of Melbourne, St. Vincent's Hospital Melbourne Victoria Australia; ^20^ University Hospital, Hotel‐Dieu Nantes France; ^21^ Baylor University Medical Center Dallas Texas USA; ^22^ University of Maryland, Greenebaum Comprehensive Cancer Center Baltimore Maryland USA; ^23^ Simmons Comprehensive Cancer Center UT Southwestern Medical Center Dallas Texas USA; ^24^ University of Calgary Charbonneau Cancer Research Institute Calgary Alberta Canada; ^25^ CHU Lille Service des Maladies du Sang F‐59000 Lille France; ^26^ Hospital Universitario de Salamanca Salamanca Spain; ^27^ Seràgnoli Institute of Hematology Bologna University School of Medicine Bologna Italy; ^28^ Karyopharm Therapeutics Inc. Newton Massachusetts USA; ^29^ Medical University of Silesia Katowice Poland; ^30^ Dana Farber Cancer Institute Boston Massachusetts USA

## Abstract

In the phase 3 BOSTON study, patients with multiple myeloma (MM) after 1–3 prior regimens were randomized to once‐weekly selinexor (an oral inhibitor of exportin 1 [XPO1]) plus bortezomib‐dexamethasone (XVd) or twice‐weekly bortezomib‐dexamethasone (Vd). Compared with Vd, XVd was associated with significant improvements in median progression‐free survival (PFS), overall response rate (ORR), and lower rates of peripheral neuropathy, with trends in overall survival (OS) favoring XVd. In BOSTON, 141 (35.1%) patients had MM with high‐risk (presence of del[17p], t[4;14], t[14;16], or ≥4 copies of amp1q21) cytogenetics (XVd, *n* = 70; Vd, *n* = 71), and 261 (64.9%) exhibited standard‐risk cytogenetics (XVd, *n* = 125; Vd, *n* = 136). Among patients with high‐risk MM, median PFS was 12.91 months for XVd and 8.61 months for Vd (HR, 0.73 [95% CI, (0.4673, 1.1406)], *p* = 0.082), and ORRs were 78.6% and 57.7%, respectively (OR 2.68; *p* = 0.004). In the standard‐risk subgroup, median PFS was 16.62 months for XVd and 9.46 months for Vd (HR 0.61; *p* = 0.004), and ORRs were 75.2% and 64.7%, respectively (OR 1.65; *p* = 0.033). The safety profiles of XVd and Vd in both subgroups were consistent with the overall population. These data suggest that selinexor can confer benefits to patients with MM regardless of cytogenetic risk. ClinicalTrials.gov identifier: NCT03110562.

## 1 INTRODUCTION

Front‐line treatment of multiple myeloma (MM) includes immunomodulatory drugs (IMiDs), monoclonal antibodies (mAbs), and/or proteasome inhibitors (PI), with or without high dose alkylating agents followed by stem cell transplantation. Despite these highly active agents, essentially all patients will relapse and require subsequent therapies. Multiple myeloma cytogenetics have been shown to influence disease outcomes, with high‐risk anomalies such as del(17p), t(4;14), t(14;16), and amp1q21^1^, ^2^, ^3^ associated with shorter progression‐free survival (PFS) and overall survival (OS) relative to those with standard‐risk cytogenetics.^4^, ^5^ Given that patients with high‐risk cytogenetics have inferior outcomes, there is a need to evaluate whether novel treatment regimens can overcome the negative impact of these cytogenetic alterations in patients with previously treated MM.

Exportin 1 (XPO1), an oncoprotein overexpressed in various hematologic and solid tumor malignancies including MM,^6^, ^7^ transports certain proteins and RNAs from the nucleus to the cytoplasm.^8^, ^9^ In addition to correlating with more aggressive MM, elevated levels of XPO1 have been shown to correlate with resistance to proteasome inhibitors^8^, ^9^, ^10^, ^11^ and to IMiDs.^12^ In cancer cells, overexpression of XPO1 leads to the nuclear export of tumor suppressor proteins (TSP) and the glucocorticoid receptor, culminating in their functional inactivation. High XPO1 also facilitates the nuclear export and translation of several oncoprotein mRNAs (e.g., cyclin D1, c‐myc) leading to elevated oncoprotein levels.^8^, ^9^, ^13^

Selinexor is a potent, oral, selective inhibitor of nuclear export (SINE) compound that binds reversibly and selectively to Cys528 in the cargo‐binding pocket of XPO1.^14^ XPO1 inhibition forces the nuclear localization and functional inactivation of TSP, trapping IκBα in the nucleus leading to suppression of nuclear factor κB (NF‐κB) activity, enhanced glucocorticoid receptor function and reduction in oncoprotein mRNA translation.^10^, ^15^ These actions induce the apoptosis of tumor cells, while largely sparing normal cells.^10^, ^16^ In preclinical studies, selinexor increased p53 localization in the nucleus and synergized with PIs to induce significant cytotoxicity in PI‐resistant MM cells^10^ and in a MM xenograft mouse model.^17^ The phase 1b/2 STOMP study confirmed these data, with XVd demonstrating on ORR of 84% in patients with bortezomib non‐refractory MM and 43% for patients with disease refractory to a PI in a previous line of therapy.^18^ This was particularly compelling, as the XVd regimen utilized only once weekly bortezomib rather than the standard twice weekly bortezomib dosing, along with 25% less dexamethasone than standard Vd. Of note, two of the four patients with high‐risk cytogenetics at screening responded to treatment with a CR in a patient with t(4;14) for 16.7 months and a VGPR in a patient with del(17p) for 32.9 months. Rates of peripheral neuropathy were low, consistent with the weekly bortezomib.

Selinexor is approved in the United States for the treatment of patients with previously treated MM^19^ and diffuse large B‐cell lymphoma.^20^ The approval for MM was based on efficacy and safety in a prespecified subgroup analysis of 83 patients in Part 2 of the STORM study (NCT02336815), in which twice weekly sel‐dex was administered to patients whose disease was refractory to bortezomib, carfilzomib, lenalidomide, pomalidomide, and daratumumab. The ORR was 25.3% with one stringent complete response (CR), four very good partial responses, and 16 partial responses.^7^ In the phase 3 BOSTON study, treatment with the once weekly selinexor plus once weekly bortezomib and low dose (40 mg) dexamethasone (XVd) was compared with standard twice‐weekly bortezomib and moderate dose (80 mg) dexamethasone (Vd) in patients with previously treated MM.^19^ Overall, relative to Vd‐treated patients, XVd‐treated patients experienced significant improvements in median PFS (13.93 vs. 9.46 months; HR 0.70, *p* = 0.0075) and ORR (76.4% vs. 62.3%), with lower rates of any‐grade (32.3% vs. 47.1%) and grade ≥2 (21.0% vs. 34.3%) peripheral neuropathy,^19^ with 37% fewer clinic visits required on the weekly XVd regimen. To further evaluate the impact of cytogenetics on the efficacy and safety of selinexor‐based combination therapy, this report summarizes outcomes from pre‐specified analyses of patients with high‐risk versus standard‐risk cytogenetics participating in the BOSTON study.

## 2 PATIENTS AND METHODS

### 2.1 Study design and patients

BOSTON (NCT03110562) was an international, active‐controlled, open‐label, randomized, phase 3 study.^19^ Eligible patients were aged ≥18 years with histologically confirmed MM per International Myeloma Working Group (IMWG) criteria^21^ previously treated with 1–3 anti‐MM regimens. Prior treatment with a PI (alone or in combination) was permitted provided that the patient had achieved at least a partial response (PR), discontinuation was not due to a treatment‐related grade ≥3 toxicity, and ≥6 months had elapsed since last dose. Patients with an Eastern Cooperative Oncology Group performance status score >2; inadequate liver, renal, or hematopoietic function; active, unstable cardiovascular function; major surgery in the 4 weeks prior to the first dose of study treatment; a history of malignancy that required treatment or showed evidence of recurrence (PI refractory disease was excluded); uncontrolled active infection; systemic light chain amyloidosis; MM characterized by central nervous system involvement; and >grade 2 peripheral neuropathy or grade ≥2 peripheral neuropathy with pain were ineligible.

BOSTON was conducted in compliance with the Declaration of Helsinki, the International Conference on Harmonization Guidelines for Good Clinical Practice, and applicable national and local regulatory requirements. The study protocol was approved by the Independent Ethics Committee/Institutional Review Board at each participating site, and all patients provided written informed consent.

### 2.2 Treatment

Patients in each participating country were randomized (1:1) to either XVd or Vd, with randomization stratified by prior PI treatment (yes vs. no), number of prior anti‐MM regimens (one vs. 2–3), and disease stage per the revised International Staging System (R‐ISS; I–II vs. III). Those assigned to XVd received oral selinexor 100 mg on Days 1, 8, 15, 22, and 29; subcutaneous bortezomib 1.3 mg/m^2^ on Days 1, 8, 15, and 22; and oral dexamethasone 20 mg on Days 1, 2, 8, 9, 15, 16, 22, 23, 29, and 30 of each 35‐day cycle. During cycles 1–8, patients assigned to Vd received subcutaneous bortezomib 1.3 mg/m^2^ on Days 1, 4, 8, and 11 and oral dexamethasone 20 mg on Days 1, 2, 4, 5, 8, 9, 11, and 12 of each 21‐day cycle. Beginning with cycle 9, patients assigned to Vd received bortezomib on Days 1, 8, 15, and 22 and dexamethasone on Days 1, 2, 8, 9, 15, 16, 22, 23, 29, and 30 of each 35‐day cycle. Treatment was administered until disease progression, unacceptable toxicity, or another discontinuation criterion was met. Patients on the Vd arm who developed objective, IRC‐confirmed progression were permitted to cross over to a selinexor‐containing regimen.

### 2.3 Outcomes

The primary endpoint was PFS (time from randomization to disease progression or death, whichever occurred first), as assessed by an independent review committee (IRC based on IMWG Criteria^21^). Secondary endpoints included OS (time from randomization to death or loss to follow‐up), ORR (determined by IRC based on IMWG criteria), duration of response (DOR; time from first confirmed response [≥PR] to confirmed disease progression or death, whichever occurred first), time to next therapy (TTNT; time from last dose of study treatment to first dose of non‐study treatment), rates of grade ≥2 peripheral neuropathy and safety. Adverse events (AEs) were coded per the Medical Dictionary for Regulatory Activities version 22.0 and graded per Common Terminology Criteria for Adverse Events version 4.03.

In pre‐specified analyses, the primary and secondary endpoints were evaluated in the subgroups of patients with high‐risk and standard‐risk cytogenetic features. Fluorescent in situ hybridization (FISH) was performed centrally on CD138‐positive cells isolated from bone marrow aspirates collected at screening. The high‐risk group included patients with at least one of the following cytogenetic abnormalities: del(17p), t(4;14), t(14;16), or amplification (≥4 copies) of 1q21 in at least 10% of screened plasma cells. Prognostic values of poor outcomes determined by FISH include cell positivity levels of 10%–20% for t(4;14) and t(14:16),^22^, ^23^ 50% for del(17p),^24^ and 20% for amp1q21.^24^, ^25^ The standard‐risk group consisted of all other patients with known or unknown baseline cytogenetics.

### 2.4 Statistics

All efficacy analyses were performed on the intent‐to‐treat (ITT) population (all randomized patients). The safety population (all patients who received ≥1 dose of study treatment) was used for the safety analyses. PFS, OS, DOR, and TTNT were evaluated using the Kaplan–Meier method. The hazard ratios (HRs) and corresponding 95% confidence intervals (CIs) were estimated via a Cox proportional‐hazards model, with treatment as a covariate. Median survival/duration and associated 95% CIs were estimated via the Kaplan–Meier method. ORR was summarized using odds ratios (ORs) and 95% CIs. The differences between treatment arms in each cytogenetic risk subgroup were compared using either a one‐sided, log‐rank test (PFS, OS, DOR, and TTNT) or a one‐sided, Cochran–Mantel–Haenszel chi‐square test (ORR). Safety outcomes were summarized using descriptive statistics. As the BOSTON study was not powered for these pre‐specified subgroup analyses, one‐sided *p* values are used throughout and should be considered nominal (i.e., for illustrative purposes only).

## 3 RESULTS

### 3.1 Patients

A total of 402 patients with MM previously treated with 1–3 prior regimens were randomized in the BOSTON study (XVd, *n* = 195; Vd, *n* = 207). Of these, 141 (35.1%) patients had MM with high‐risk cytogenetics (XVd, *n* = 70; Vd, *n* = 71) and 261 (64.9%) had MM with standard‐risk cytogenetics (XVd, *n* = 125; Vd, *n* = 136). Baseline demographic and disease characteristics were generally balanced between treatment arms in subgroups defined by cytogenetic risk (Table 1). In both treatment arms, amplification of 1q21 (≥4 copies) was the most common cytogenetic abnormality (XVd, 22.1%; Vd, 18.8%) (Table S1). Proportionally fewer males than females had high‐risk cytogenetic features (51.8% [73/141] vs. 60.2% [157/261]). Bortezomib was the most commonly used prior therapy (high‐risk, 75.2% [106/141]; standard‐risk, 66.3% [173/261]) followed by lenalidomide (high‐risk, 39.0% [55/141]; standard‐risk, 37.9% [99/261]).

**TABLE 1:** Baseline characteristics by cytogenetic risk status and treatment

	High‐risk cytogenetics^a^		Standard‐risk cytogenetics	
	XVd (*n* = 70)	Vd (*n* = 71)	XVd (*n* = 125)	Vd (*n* = 136)
Median age, years (range)	70 (45–84)	71 (49–90)	65 (40–87)	67 (38–84)
Age group, *n* (%)				
18–50 years	4 (5.7)	1 (1.4)	11 (8.8)	10 (7.4)
51–64 years	24 (34.3)	23 (32.4)	47 (37.6)	41 (30.1)
65–74 years	29 (41.4)	32 (45.1)	46 (36.8)	53 (39.0)
≥75 years	13 (18.6)	15 (21.1)	21 (16.8)	32 (23.5)
Male, *n* (%)	34 (48.6)	39 (54.9)	81 (64.8)	76 (55.9)
ECOG performance status, *n* (%)				
0	29 (41.4)	28 (39.4)	40 (32.0)	49 (36.0)
1	34 (48.6)	41 (57.7)	72 (57.6)	73 (53.7)
2	7 (10.0)	2 (2.8)	13 (10.4)	14 (10.3)
ISS stage at screening, *n* (%)				
I	37 (52.9)	34 (47.9)	60 (48.0)	68 (50.0)
II	21 (30.0)	26 (36.6)	45 (36.0)	47 (34.6)
III	12 (17.1)	11 (15.5)	20 (16.0)	21 (15.4)
R‐ISS disease stage at screening, *n* (%)				
I	10 (14.3)	8 (11.3)	46 (36.8)	44 (32.4)
II	53 (75.7)	53 (74.6)	64 (51.2)	72 (52.9)
III	6 (8.6)	8 (11.3)	6 (4.8)	8 (5.9)
Unknown	1 (1.4)	2 (2.8)	9 (7.2)	12 (8.8)
Median time since initial diagnosis, years (range)	3.5 (1.1–23.0)	3.0 (0.6–22.0)	4.08 (0.4–21.5)	3.83 (0.4–18.4)
Lines of prior therapy, *n* (%)				
1	35 (50.0)	32 (45.1)	64 (51.2)	67 (49.3)
2	22 (31.4)	20 (28.2)	43 (34.4)	44 (32.4)
3	13 (18.6)	19 (26.8)	18 (14.4)	25 (18.4)
Prior SCT, *n* (%)	26 (37.1)	28 (39.4)	50 (40.0)	35 (25.7)
Prior therapy, *n* (%)				
Bortezomib	54 (77.1)	52 (73.2)	80 (64.0)	93 (68.4)
Carfilzomib	11 (15.7)	9 (12.7)	9 (7.2)	12 (8.8)
Ixazomib	3 (4.3)	1 (1.4)	3 (2.4)	2 (1.5)
Daratumumab	3 (4.3)	5 (7.0)	8 (6.4)	1 (0.7)
Lenalidomide	26 (37.1)	29 (40.8)	51 (40.8)	48 (35.3)
Pomalidomide	4 (5.7)	3 (4.2)	7 (5.6)	4 (2.9)

Abbreviations: ECOG, Eastern Cooperative Oncology Group; ISS, International Staging System; R‐ISS, Revised International Staging System; SCT, stem cell transplantation; Vd, bortezomib and dexamethasone; XVd, selinexor, bortezomib, and dexamethasone.

^a^ Patients were considered high‐risk if they presented with ≥1 of the following cytogenetic abnormalities: del17p, t(4;14), t(14;16), or amplification of 1q21 (≥4 copies).

### 3.2 Efficacy

**FIGURE 1:**
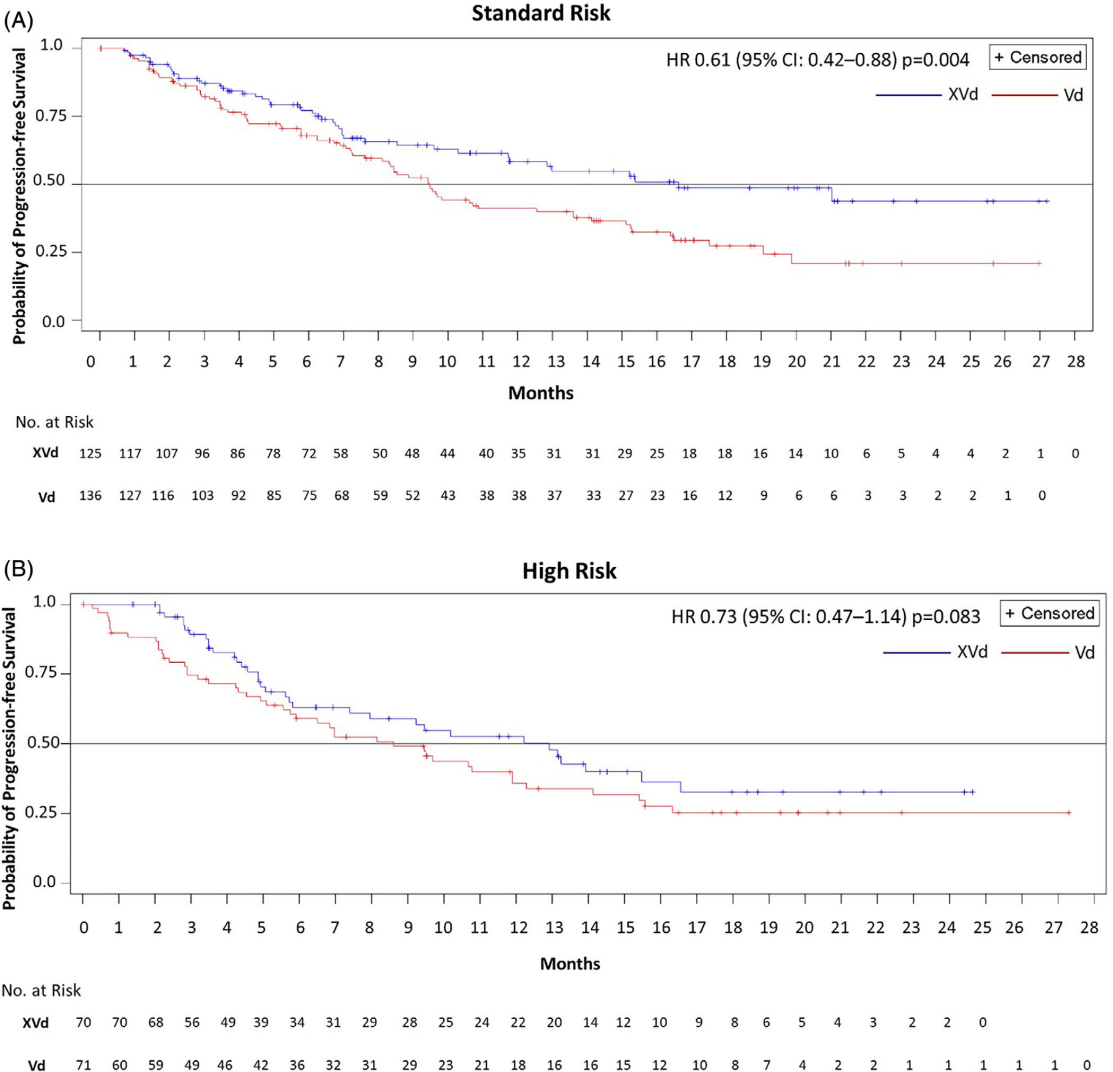
Progression‐free survival. Patients with A, high‐risk and B, standard‐risk cytogenetics. CI, confidence interval; HR, hazard ratio; PFS, progression‐free survival; Vd, bortezomib and dexamethasone; XVd, selinexor, bortezomib, and dexamethasone [Color figure can be viewed at wileyonlinelibrary.com]

Median PFS in the high‐risk group was longer for patients who received XVd (12.91 months) versus 8.61 months in those that received Vd (HR, 0.73; 95% CI, 0.47–1.14; one‐sided *p* = 0.083) (Figure 1(A)). Among those with standard‐risk cytogenetics, median PFS was longer for XVd than for Vd: 16.62 versus 9.46 months (HR, 0.61; 95% CI, 0.42–0.88; one‐sided *p* = 0.004) (Figure 1(B)).

The ORR was greater for XVd than for Vd in both the high‐risk (78.6% vs. 57.7%; one‐sided *p* = 0.004) and standard‐risk (75.2% vs. 64.7%; *p* = 0.033) subgroups (Table 2). In the high‐risk subgroup, the proportion of patients who achieved a very good PR (VGPR) or better was almost two‐fold higher for XVd than for Vd (30.0% vs. 18.3%). Patients receiving XVd in both standard‐risk and high‐risk groups had had lower progressive disease (PD) rates than patients receiving Vd (high‐risk, XVd 0% vs. Vd 7.0%; standard‐risk. XVd 0.8% vs. Vd 3.7%). The rate of stable disease (SD) in high‐risk patients was lower in the XVd arm (7.1%) as compared to the Vd arm (21.1%). Median TTNT was prolonged with XVd relative to Vd in both the high‐risk (14.03 vs. 8.61 months; one‐sided *p* = 0.018) and standard‐risk (18.23 vs. 11.73 months; one‐sided *p* = 0.018) subgroups (Table 2).

**TABLE 2:** Efficacy outcomes by cytogenetic risk status and treatment

	High‐risk cytogenetics^a^		Standard‐risk cytogenetics	
	XVd (*n* = 70)	Vd (*n* = 71)	XVd (*n* = 125)	Vd (*n* = 136)
Median PFS	12.91	8.61	16.62	9.46
HR (95% CI)	0.73 (0.47–1.14)		0.61 (0.42–0.88)	
One‐sided *p* value	0.083		0.004	
Median OS, months	22.87	24.84	NR	NR
HR (95% CI)	0.87 (0.52–1.46)		0.75 (0.46–1.23)	
One‐sided *p* value	0.304		0.129	
ORR, *n* (%)	55 (78.6)	41 (57.7)	94 (75.2)	88 (64.7)
OR (95% CI)	2.68 (1.28–5.62)		1.65 (0.97–2.83)	
One‐sided *p* value	0.004		0.033	
Best overall response, *n* (%)				
Stringent CR	4 (5.7)	3 (4.2)	15 (12.0)	10 (7.4)
≥CR	4 (5.7)	5 (7.0)	10 (8.0)	4 (2.9)
VGPR	21 (30.0)	13 (18.3)	33 (26.4)	32 (23.5)
PR	26 (37.1)	20 (28.2)	36 (28.8)	42 (30.9)
MR	10 (14.3)	5 (7.0)	6 (4.8)	15 (11.0)
SD	5 (7.1)	15 (21.1)	20 (16.0)	25 (18.4)
PD	0	5 (7.0)	1 (0.8)	5 (3.7)
NE	0	5 (7.0)	4 (3.2)	3 (2.2)
Median DOR, months	12.55	12.68	NR	12.88
HR (95% CI)	1.04 (0.59–1.83)		0.61 (0.38–0.98)	
One‐sided *p* value	0.55		0.019	
Median TTNT, months	14.03	8.61	18.23	11.73
HR (95% CI)	0.64 (0.42–0.97)		0.71 (0.51–0.98)	
One‐sided *p* value	0.018		0.018	

Abbreviations: CI, confidence interval; CR, complete response; DOR, duration of response; HR, hazard ratio; MR, minimal response; NE, not evaluated; OR, odds ratio; ORR, overall response rate; OS, overall survival; PD, progressive disease; PR, partial response; SD, stable disease; TTNT, time to next treatment; Vd, bortezomib and dexamethasone; VGPR, very good partial response; XVd, selinexor, bortezomib, and dexamethasone.

^a^ Patients were considered high‐risk if they presented with ≥1 of the following cytogenetic abnormalities: del17p, t(4;14), t(14;16), or amplification of 1q21 (≥4 copies).

Despite patients in the trial being relatively early in their course of disease, the limited follow up (median around 21 months for all groups), and the ability for patients on Vd with objective progression to cross over to a selinexor containing regimen, the OS in the high‐ and standard‐risk subgroups tended to favor XVd: In the high‐risk subgroup, the HR for OS was 0.87 (XVd vs. Vd, 95% CI [0.52, 1.46], one‐sided *p* = 0.304). In the standard‐risk subgroup, the HR for OS was 0.75 (95% CI [0.46, 1.23], one‐sided *p* = 0.129). Patients treated with XVd had numerically lower rates of death in both the standard risk group (21.6%) and in the high‐risk group (38.6%) as compared to Vd treated patients (28.7% and 43.7% respectively), although these trends did not reach statistical significance.

Efficacy endpoints were also evaluated by type of cytogenetic abnormality (Table S2). The results in patients with del(17p), that is, deletion of at least one copy of the p53 TSP, demonstrated a significant increase in PFS with XVd treatment (12.22 vs. 5.91 months; HR, 0.38; 95% CI, 0.16–086; one‐sided *p* = 0.008). ORR was significantly also improved with XVd compared to Vd: 76.2% vs. 37.5% (OR 5.3, CI95% 1.28–22.19; one‐sided *p* = 0.010), as well as median TTNT: 14.78 versus 7.62 months (HR 0.30 95% CI, 0.12–0.75; one‐sided *p* = 0.003). Patients with del(17p) treated with XVd versus Vd had a trend towards improved DOR (14.75 vs. 6.82 months; HR 0.43, 95% CI 0.13–1.40; one‐sided *p* = 0.08). Interestingly, the OS in patients with del(17p) significantly favored XVd: HR 0.43 (95% CI 0.16–1.16; one‐sided *p* = 0.04), although the median values were similar: 22.21 versus 21.22 months. In the subset of patients with two or more cytogenetic abnormalities, a trend towards improved ORR was observed with XVd compared to Vd: 85.0% versus 64.7% (OR 3.09, 95% CI 0.64–15.00; one‐sided *p* = 0.079). While standard risk patients demonstrated significantly prolonged PFS with XVd, PFS in patients with ≥2 cytogenetic abnormalities receiving XVd (15.47 vs. 5.91 months; HR 0.54, 95%CI 0.22–1.33; one‐sided *p* = 0.09) and TTNT (14.03 vs. 7.62 months; HR 0.54, 95% CI 0.23–1.30; one‐sided *p* = 0.082) (Table S3) showed a trend in favor of XVd over Vd.

Generally, XVd conferred improvements in median PFS and ORR relative to Vd in patient subgroups defined by cytogenetic risk status except t(14;16) and baseline disease characteristics, including number of prior lines of therapy, prior treatment with lenalidomide, and creatinine clearance.^19^, ^26^ Interestingly, XVd was significantly more effective over Vd for patients with R‐ISS Stages I‐II disease (Table S4).

### 3.3 Safety

In the high‐risk and standard‐risk subgroups, one and two patients, respectively, assigned to Vd did not receive study treatment. Thus, the safety analyses included 70 XVd‐treated and 70 Vd‐treated patients with high‐risk cytogenetics and 125 XVd‐treated and 134 Vd‐treated patients with standard‐risk cytogenetics. The safety profiles of XVd and Vd in the high‐risk and standard‐risk subgroups were generally similar. Over the course of the therapy (average duration e_x007E;10 months), the majority of patients in each subgroup (94.3%–100.0%) experienced ≥1 AE (Table 3). In both cytogenetic risk subgroups, thrombocytopenia and nausea were the most common AEs and were more frequently reported in XVd‐ versus Vd‐treated patients.

**TABLE 3:** Safety outcomes by cytogenetic risk status and treatment

	High‐risk cytogenetics^a^		Standard‐risk cytogenetics	
	XVd (*n* = 70)	Vd (*n* = 70)	XVd (*n* = 125)	Vd (*n* = 134)
Any AE, *n* (%)^b^	70 (100.0)	66 (94.3)	124 (99.2)	132 (98.5)
Thrombocytopenia	50 (71.4)	23 (32.9)	70 (56.0)	33 (24.6)
Nausea	34 (48.6)	5 (7.1)	64 (51.2)	15 (11.2)
Fatigue	30 (42.9)	14 (20.0)	52 (41.6)	23 (17.2)
Neuropathy peripheral	26 (37.1)	34 (48.6)	37 (29.6)	63 (47.0)
Decreased appetite	22 (31.4)	1 (1.4)	47 (37.6)	10 (7.5)
Anemia	21 (30.0)	16 (22.9)	51 (40.8)	32 (23.9)
Diarrhea	20 (28.6)	14 (20.0)	45 (36.0)	38 (28.4)
Weight decreased	19 (27.1)	6 (8.6)	32 (25.6)	19 (14.2)
Asthenia	19 (27.1)	7 (10.0)	29 (23.2)	20 (14.9)
Upper respiratory tract infection	14 (20.0)	10 (14.3)	25 (20.0)	20 (14.9)
Cataract	11 (15.7)	5 (7.1)	32 (25.6)	9 (6.7)
Vomiting	11 (15.7)	0	29 (23.2)	10 (7.5)
Grade ≥3 hematologic AEs, *n* (%)^c^	60 (85.7)	45 (64.3)	106 (84.8)	82 (61.2)
Thrombocytopenia	38 (54.3)	14 (20.0)	40 (32.0)	22 (16.4)
Anemia	10 (14.3)	7 (10.0)	21 (16.8)	14 (10.4)
Neutropenia	9 (12.9)	5 (7.1)	8 (6.4)	2 (1.5)
Grade ≥3 non‐hematologic AEs, *n* (%)^c^				
Pneumonia	11 (15.7)	9 (12.9)	11 (8.8)	13 (9.7)
Fatigue	10 (14.3)	0	16 (12.8)	2 (1.5)
Peripheral neuropathy	5 (7.1)	3 (4.3)	4 (3.2)	15 (11.2)
Asthenia	5 (7.1)	3 (4.3)	11 (8.8)	6 (4.5)
Cataract	6 (8.6)	1 (1.4)	13 (10.4)	3 (2.2)
Nausea	4 (5.7)	0	11 (8.8)	0
Hypertension	3 (4.3)	3 (4.3)	5 (4.0)	3 (2.2)
Diarrhea	2 (2.9)	0	11 (8.8)	1 (0.7)
Hypophosphatemia	3 (4.3)	1 (1.4)	7 (5.6)	2 (1.5)
Hyponatraemia	2 (2.9)	0	7 (5.6)	1 (0.7)
Vomiting	0	0	8 (6.4)	0
Decreased appetite	0	0	7 (5.6)	0
Discontinuation due to an AE, *n* (%)	11 (15.7)	6 (8.6)	30 (24.0)	26 (19.4)
Death due to an AE, *n* (%)	2 (2.9)	3 (4.3)	10 (8.0)	7 (5.2)

Abbreviations: AE, adverse event; Vd, bortezomib and dexamethasone; XVd, selinexor, bortezomib, and dexamethasone.

^a^ Cytogenetic risk status was determined centrally via fluorescent in situ hybridization.

^b^ Preferred terms reported in >20% of patients in any subgroup are presented.

^c^ Preferred terms reported in >5% of patients in any subgroup are presented.

The most common grade ≥3 AE was thrombocytopenia, which was more common in XVd‐treated than Vd‐treated patients in both the high‐risk (54.3% vs. 20.0%) and standard‐risk (32.0% vs. 16.4%) subgroups (Table 3). Of note, despite the higher rates of thrombocytopenia with XVd versus Vd, the occurrence of clinically significant (Grade ≥3) bleeding was similar and low (i.e., only one in XVd and two in Vd). In the high‐risk subgroup, the rate of grade ≥2 peripheral neuropathy was less common in XVd‐treated than in Vd‐treated patients (25.7% vs. 35.7%). Similarly, in the standard‐risk subgroup, the rate of grade ≥2 peripheral neuropathy was lower with XVd than Vd (18.4% vs. 33.6%). The most common non‐hematologic grade ≥3 AE was pneumonia, which occurred at similar frequencies across treatments arms in the high‐risk (12.9%–15.7%) and standard‐risk (9.7%–8.8%) subgroups.

The proportion of patients who discontinued treatment due to an AE was greater among XVd‐treated than Vd‐treated patients in the high‐risk subgroup (15.7% vs. 8.6%) and in the standard‐risk subgroup (24.0% vs. 19.4%). Five and 17 patients in the high‐risk (XVd, *n* = 2; Vd, *n* = 3) and standard‐risk (XVd, *n* = 10; Vd, *n* = 7) subgroups, respectively, died due to an AE.

## 4 DISCUSSION

The BOSTON trial represents the first large Phase 3 study in previously treated MM to utilize once weekly bortezomib and low dose dexamethasone in the experimental arm, consistent with common clinical practice. The once weekly XVd regimen demonstrated superior PFS, ORR, and TTNT, with a trend towards improved OS, as compared with standard twice weekly Vd across the entire population of patients enrolled in the study. In the pre‐specified subgroup analyses from the phase 3 BOSTON study, XVd yielded more favorable efficacy outcomes relative to Vd in patients with at least one prior MM therapy irrespective of cytogenetic risk status. These results become more striking when one considers that XVd‐treated patients received 40% less bortezomib and 25% less dexamethasone, with ~37% fewer clinic visits, than Vd‐treated patients during the first 24 weeks of the study. Moreover, the use of weekly bortezomib in the XVd arm reflects the more common clinical practice when using bortezomib‐based triplets. XVd treatment significantly improved PFS by 7 months compared to Vd in standard‐risk patients, while a trend was observed in the high‐risk subgroup, as XVd‐treated patients tended to have longer median PFS duration compared to those receiving Vd. Despite the limitation of the cross‐trial comparison, results compare favorably with subgroup analyses of two phase 3 studies of patients with previously treated MM: CASTOR and OPTIMISSM, both of which used higher doses of Vd on the experimental arms.

In CASTOR, patients were randomized to receive daratumumab (anti‐CD38), twice weekly bortezomib, and moderate dose dexamethasone (DVd) or Vd,^27^ but the bortezomib/dexamethasone was discontinued on both arms after 24 weeks. Among DVd‐treated patients, the median PFS duration was 12.6 months in the high‐risk subgroup and 16.6 months in the standard‐risk subgroup.^27^ Of note, the CASTOR subgroup analyses only regarded del(17p), t(4;14), and t(14;16) as high‐risk cytogenetic abnormalities.^27^ The present analysis of BOSTON also included amplification of 1q21, which is notable as the presence of ≥3 copies of 1q21 has been demonstrated to be negatively prognostic of response to bortezomib‐based treatment and may confer resistance to bortezomib.^28^ Here, we focused our analyses on the more conservative definition of ≥4 copies of 1q21 as high risk.^29^ Of particular note, overall rates of PN on DVd were 49% and 55% in standard‐ and high‐risk patients, respectively, substantially higher than the rates on the Vd arm in that study.^27^

In OPTIMISMM, patients with relapsed or refractory MM who had received 1–3 previous regimens (including two or more cycles of lenalidomide) and had progressive disease were randomly assigned 1:1 to pomalidomide, *twice weekly* bortezomib, and dexamethasone (PVd) or Vd.^30^ As in the CASTOR trial, patients were considered to be high risk if they had one of the following abnormalities: del(17p), t(4;14), or t(14;16). In this specified subgroup, median PFS was lower than that in the ITT population; however, patients receiving PVd had an improved PFS of 8.44 months as compared to 5.32 months in Vd‐treated subjects (HR 0.56, 95% CI 0.35–0.90, *p* = 0.021). In BOSTON, the PFS benefit observed in XVd‐treated patients was most evident among those with del(17p), t(4;14), and amplification of 1q21 (≥4 copies). Only the patients with t(14;16) trended more poorly on XVd versus Vd with a PFS HR of 1.46 and an overlapping 95% CI (0.45–4.80) with a one‐sided *p* > 0.05. In contrast, the ORR in this subgroup was numerically higher (85%) in the XVd arm as compared to the Vd arm (55%). These disparate results may have been due to the small population of patients with t(14;16) MM in the study, and will be evaluated in future studies. In terms of ORR, deeper responses (≥VGPR) were observed in XVd‐treated versus Vd‐treated patients with either high‐risk (30.0% [21/70] vs. 18.3% [13/71]) or standard‐risk (26.4% [33/125] vs. 23.5% [32/136]) cytogenetics. Moreover, the impact of XVd on efficacy outcomes did not appear to be negatively affected by the number of cytogenetic abnormalities or most baseline disease characteristics.

The safety profiles of XVd and Vd in the high‐risk and standard‐risk subgroups were similar to each other and consistent with those observed in the overall study population.^19^ As in the primary analysis of BOSTON,^19^ the most common grade ≥3 AE reported in the high‐risk and standard‐risk subgroups was thrombocytopenia. Importantly, rates of PN were lower in all XVd cohorts as compared with the Vd cohorts. While discontinuation rates were higher with XVd due to AEs, a significantly prolonged TTNT was observed suggesting that these patients were responding well and did not require immediate salvage therapy in contrast to patients who discontinued. Furthermore, improved supportive care as a result of physician experience is anticipated to further enhance the activity of XVd, and lead to a reduction in the number of patients discontinuing therapy due to AEs.

Preclinically, the combination of selinexor with a PI shows remarkable synergy.^17^, ^31^, ^32^ Mechanistically, XPO1 inhibition forces the nuclear retention and functional activation of TSPs, and PIs prevent both the nuclear and cytoplasmic degradation of TSPs including IκB (and other proteins), leading to markedly higher levels of TSPs and reduced NFκB activity in the nucleus of cells treated with the combination as compared with the individual components.^31^, ^33^ With about 20 major TSPs, cells with abnormalities in one (e.g., del(17p) affecting p53 levels) or several of these proteins may be induced to undergo apoptosis via activation of remaining, wild type TSPs. Furthermore, the combination of selinexor with dexamethasone induces synergy on several levels: retention of transcriptionally active glucocorticoid receptor in the nucleus, induction of glucocorticoid anti‐proliferative expression and activity leading to the inhibition of the mTOR pathway and to enhanced MM cancer cell death.^32^

Changes in gene expression that support cell proliferation and tumorigenesis have been observed in MM with any of the four high‐risk cytogenetics analyzed in this study. Given the novel mechanism of action against a single target that impacts most TSP pathways, we hypothesize that these high‐risk associated changes would still be inhibited by selinexor. For example, del(17p) results in loss of the TSP p53 expression,^34^ but the loss of p53 by (using siRNA knockdown) did not reduce sensitivity to selinexor.^35^ Translocation of t(4:14) upregulates fibroblast growth factor receptor 3 (FGFR3),^36^ which activates mitogenic pathways including AKT, MAP mTOR and NFκB – all inhibited by selinexor.^37^ The t(4;14) and t(14:16) translocation may lead to the overexpression of the c‐MAF protooncogene that upregulates IL‐4 and IL‐10 cytokine expression,^38^ and selinexor may negate such transactivation through the inhibition of the NFKB signaling.^39^ Lastly, the amp1q21 may result in the overexpression of CKS1B, upregulating cyclins and CDK activity, overcoming normal cell‐cycle checkpoints that are activated by the inhibition of XPO1 with selinexor.^37^, ^40^ Taken together, despite the relatively small sample size for each cytogenetic abnormality, our results show significant differences based on high‐risk cytogenetic profiles that also align with the potential mechanism of action.

BOSTON enrolled one of the greatest numbers of clinical trial participants with high‐risk cytogenetics as compared to the other large bortezomib‐based randomized studies.^27^, ^30^ However, even though the analyses in this subpopulation were pre‐specified, a major limitation of the present analysis is the fact that BOSTON was not powered statistically to compare outcomes between patients with high‐risk and standard‐risk cytogenetics. While XVd improved outcomes compared to Vd in high‐risk patients, the confidence intervals over suggest that standard‐risk patients may have had more significant improvements. In addition, no adjustment was made for multiplicity testing, and the cytogenetic risk status was unknown for 10.0% of patients. Despite the plethora of additional therapies available to patients following progression on BOSTON, and allowing crossover from Vd to a selinexor‐based regimen on objective progression, a trend towards improved OS for XVd over Vd was also observed, suggesting that treatment with selinexor may have modified the biology of the MM. However, given the limitations noted above, these results should be considered hypothesis‐generating.

In conclusion, these data suggest that the oral XPO1 inhibitor selinexor, with its novel mechanism of action, can be used to treat patients with previously treated MM with either high‐risk or standard‐risk cytogenetics. XVd provided a non‐IMiD‐based regimen with superior benefits over Vd in a number of clinical outcomes across both the high‐risk and standard‐risk populations. Given that most patients now receive lenalidomide‐based therapy in the frontline setting, a non‐IMiD based, simple triplet regimen with reduced long‐term toxicity (e.g., peripheral neuropathy) and without the need for intravenous or prolonged subcutaneous infusions makes it a viable option in the treatment of patients with MM after at least one prior regimen, whether they have high risk or standard risk disease.

## CONFLICT OF INTEREST

Ajai Chari reports grants and personal fees from Janssen, Celgene, Novartis, Amgen, Seattle Genetics, and Millenium/Takeda; personal fees from Bristol Myers Squibb, Karyopharm, Sanofi, Oncopeptides, Antengene, Glaxo Smith Kline, Secura Bio, and Shattuch Labs.

Ivan Spicka reports personal fees from Janssen‐Cilag, Takeda, Sanofi Aventis and Novartis; personal fees and non‐financial support from Colgene, BMS and Amgen.

Iryna Kriachok reports a consulting role, an advisory role, and a speaker's bureau role for Takeda, Janssen, Roche, Abbvie and MSD; Travel support by Takeda, MSD, Roche, Abbvie and Janssen.

Holger W. Auner reports an advisory role for Takeda and Karyopharm; grant from Amgen; and a speaker's bureau role for Janssen.

Roman Hajek has had a consultant or advisory relationship with Janssen, Amgen, Celgene, AbbVie, BMS, Novartis, PharmaMar, and Takeda; has received honoraria from Janssen, Amgen, Celgene, BMS, PharmaMar, and Takeda; has received research funding from Janssen, Amgen, Celgene, BMS, Novartis, and Takeda.

Christopher P. Venner has received honoraria from BMS/Celgene, Janssen, Sanofi, Amgen, GSK, and Takeda.

Mamta Garg reports support for attending conferences from Takeda; an advisory role for Amgen, Takeda, Jansen, Novartis and Celgene; and a speaker's bureau role for Janssen.

Hang Quach reports grants from and an advisory board role for Amgen, Celgene, Karyopharm, GlaxoSmithKline; non‐financial support and research drug supply from Sanofi; an advisory board role for Janssen Cilag and Specialized therapeutics.

Sundar Jagannath reports consulting services for AbbVie, Bristol‐Myers Squibb, Janssen Pharmaceuticals, Merck & Co. PM reports personal fees from Celgene, Amgen, Takeda, Janssen and Abbvie.

Moshe Levy reports receiving consulting fees and lecture fees from Takeda, Celgene, Seattle Genetics, AbbVie, Jazz Pharmaceuticals, Gilead Sciences, Bristol‐Myers Squibb, Amgen, Spectrum Pharmaceuticals, and Janssen.

Larry D. Anderson, Jr. reports honoraria from advisory board activity from the following: GSK, Amgen, Janssen, BMS/Celgene, Karyopharm, and Oncopeptides.

Nizar J. Bahlis reports grants and personal fees from Celgene; personal fees from Janssen, Amgen, Takeda, Abbvie, GSK and Karyopharm.

Thierry Facon reports an advisory board role for Karyopharm, Amgen, Roche and Oncopeptides; an advisory board role and a speaker bureau role for Janssen, Celgene/BMS, and Takeda.

Maria Victoria Mateos has served as member of advisory boards or received honoraria from Janssen, BMS‐Celgene, Takeda, Amgen, Sanofi, Oncopeptides, GSK, Adaptive, Pfizer, Regeneron, Roche and Sea‐Gen.

Hua Chang, Yosef Landesman, Yi Chai, Melina Arazy, Jatin Shah and Michael G. Kauffman are salaried employees and stockholders of Karyopharm Therapeutics Inc.

Sharon Shacham reports being employed by and owning stock in Karyopharm Therapeutics, holding patents (8999996, 9079865, 9714226, PCT/US12/048319, and I574957) on hydrazide‐containing nuclear transport modulators and uses, and holding pending patents (PCT/US12/048319, 499/2012, PI20102724, and 2012000928) on hydrazide‐containing nuclear transport modulators and uses.

Paul G. Richardson reports receiving grant support and honoraria from Oncopeptides, Celgene, and Takeda, grant support from Bristol‐Myers Squibb, and honoraria from Amgen, Janssen, and Karyopharm Therapeutics.

## Supporting information

**Table S1**. Prevalence of cytogenetic abnormalities^*^ among participants in the BOSTON study
**Table S2**. Efficacy outcomes by type of cytogenetic abnormality and treatment
**Table S3**. Efficacy outcomes by number of cytogenetic abnormalities and treatment
**Table S4**. Efficacy outcomes by cytogenetic risk status, baseline disease characteristics, and treatment

## Data Availability

Karyopharm Therapeutics agrees to share individual participant data that underlie the results reported in this article (after deidentification), including the study protocol and statistical analysis plan. Data availability will begin 9 months after publication and will be available 36 months after publication. To gain access, data requestors should submit a proposal to medicalinformation@karyopharm.com. Proposals will be reviewed by an independent review committee identified for this purpose.
