# Supplementary material for: Selinexor, bortezomib, and dexamethasone versus bortezomib and dexamethasone in previously treated multiple myeloma: Outcomes by cytogenetic risk
Source: Am J Hematol. 2021 Jul 5;96(9):1120–30. doi: 10.1002/ajh.26261 (PMC8457116; doi:10.1002/ajh.26261)
Supplement: Supplementary file 1 — Table S1. Prevalence of cytogenetic abnormalities* among participants in the BOSTON study Table S2. Efficacy outcomes by type of cytogenetic abnormality and treatment Table S3. Efficacy outcomes by number of cytogenetic abnormalities and treatment Table S4. Efficacy outcomes by cytogenetic risk status, baseline disease characteristics, and treatment [file AJH-96-1120-s001.docx]

**Selinexor, bortezomib, and dexamethasone versus bortezomib and dexamethasone in previously treated multiple myeloma: Outcomes by cytogenetic risk**

Shambavi Richard, Ajai Chari, Sosana Delimpasi, Maryana Simonova, Ivan Spicka, Ludek Pour, Iryna Kriachok, Meletios A. Dimopoulos, Halyna Pylypenko, Holger W. Auner, Xavier Leleu,

Ganna Usenko, Roman Hajek, Reuben Benjamin, Tuphan Kanti Dolai,

Dinesh Kumar Sinha, Christopher P Venner, Mamta Garg, Don Ambrose Stevens, Hang Quach, Sundar Jagannath, Phillipe Moreau, Moshe Levy, Ashraf Badros, Larry D. Anderson, Jr.

Nizar J. Bahlis, Thierry Facon, Maria Victoria Mateos, Michele Cavo, Hua Chang,

Yosef Landesman, Yi Chai, Melina Arazy, Jatin Shah, Sharon Shacham, Michael G. Kauffman, Sebastian Grosicki, Paul G. Richardson

**Supplementary Material**

- **Table S1. Prevalence of cytogenetic abnormalities^*^ among participants in the BOSTON study**
- **Table S2. Efficacy outcomes by type of cytogenetic abnormality and treatment**
- **Table S3. Efficacy outcomes by number of cytogenetic abnormalities and treatment**
- **Table S4. Efficacy outcomes by cytogenetic risk status, baseline disease characteristics, and treatment**

**Table S1. Prevalence of cytogenetic abnormalities^*^ among participants in the BOSTON study**

|  | **XVd (n=195)** | **Vd  (n=207)** |
| --- | --- | --- |
| Cytogenetic abnormality, n (%) |  |  |
| del(17p) | 21 (10.8) | 16 (7.7) |
| t(14;16) | 7 (3.6) | 11 (5.3) |
| t(4;14) | 22 (11.3) | 27 (13.0) |
| amp1q21 (≥4 copies) | 43 (22.1) | 39 (18.8) |
| del(17p) or t(14;16) or t(4;14) or amp1q21 (≥4 copies) | 70 (35.9) | 71 (34.3) |

*Cytogenetic risk status was determined centrally via fluorescent *in situ* hybridization.
Vd, bortezomib and dexamethasone; XVd, selinexor, bortezomib, and dexamethasone.

**Table S2. Efficacy outcomes by type of cytogenetic abnormality and treatment**

|  | **del17p** | | **t(4;14)** | | **t(14;16)** | | **amp1q21 (≥4 copies)** | |
| --- | --- | --- | --- | --- | --- | --- | --- | --- |
|  | **XVd (n=21)** | **Vd (n=16)** | **XVd (n=22)** | **Vd (n=27)** | **XVd (n=7)** | **Vd (n=11)** | **XVd (n=43)** | **Vd (n=39)** |
| Median PFS, months | 12.22 | 5.91 | 13.24 | 8.33 | 4.57 | 11.89 | 12.91 | 8.15 |
| HR (95% CI) | 0.38 (0.16–0.86) | | 0.70 (0.32–1.52) | | 1.46 (0.45–4.80) | | 0.63 (0.34–1.17) | |
| One-sided p-value | 0.0080 | | 0.18 | | 0.75 | | 0.07 | |
| Median OS, months | 22.21 | 21.22 | NE | 23.49 | 15.18 | NE | 27.43 | 23.49 |
| HR (95% CI) | 0.43 (0.16–1.16) | | 0.62 (0.23–1.65) | | 2.03 (0.54–7.59) | | 0.85 (0.41–1.76) | |
| One-sided p-value | 0.04 | | 0.17 | | 0.86 | | 0.33 | |
| ORR, n (%) | 16 (76.2) | 6 (37.5) | 20 (90.9) | 20 (74.1) | 6 (85.7) | 6 (54.5) | 33 (76.7) | 24 (61.5) |
| OR (95% CI) | 5.33 (1.28–22.19) | | 3.50 (0.65–18.95) | | 5.00 (0.44–56.62) | | 2.06 (0.79–5.37) | |
| One-sided p-value | 0.0096 | | 0.07 | | 0.09 | | 0.07 | |
| Median DOR, months | 14.75 | 6.82 | 12.55 | 8.90 | 3.71 | NE | 15.34 | 12.68 |
| HR (95% CI) | 0.43 (0.13–1.40) | | 0.82 (0.35–1.89) | | 3.26 (0.69–15.36) | | 0.84 (0.39–1.81) | |
| One-sided p-value | 0.08 | | 0.32 | | 0.94 | | 0.33 | |
| Median TTNT, months | 14.78 | 7.62 | 14.13 | 12.25 | 6.93 | 13.04 | 14.06 | 7.87 |
| HR (95% CI) | 0.30 (0.12–0.75) | | 0.76 (0.35–1.63) | | 1.40 (0.47–4.20) | | 0.57 (0.33–1.01) | |
| One-sided p-value | 0.0033 | | 0.24 | | 0.73 | | 0.03 | |

CI, confidence interval; CR, complete response; DOR, duration of response; HR, hazard ratio; NR, not reached; OR, odds ratio; ORR, overall response rate; OS, overall survival; PFS, progression-free survival; TTNT, time to next treatment; Vd, bortezomib and dexamethasone; XVd, selinexor, bortezomib, and dexamethasone.

**Table S3. Efficacy outcomes by number of cytogenetic abnormalities and treatment**

|  | **1 cytogenetic abnormality^*^** | | **≥2 cytogenetic abnormalities^*^** | |
| --- | --- | --- | --- | --- |
|  | **XVd (n=48)** | **Vd (n=50)** | **XVd (n=20)** | **Vd (n=17)** |
| Median PFS, months | 10.18 | 8.61 | 15.47 | 5.91 |
| HR (95% CI) | 0.69 (0.41–1.15) | | 0.54 (0.22–1.33) | |
| One-sided p-value | 0.08 | | 0.09 | |
| Median OS, months | 27.43 | 24.84 | NE | 23.49 |
| HR (95% CI) | 0.79 (0.43–1.43) | | 0.74 (0.25–2.20) | |
| One-sided p-value | 0.21 | | 0.29 | |
| ORR, n (%) | 36 (75.0) | 26 (52.0) | 17 (85.0) | 11 (64.7) |
| OR (95% CI) | 2.77 (1.18–6.53) | | 3.09 (0.64–15.00) | |
| One-sided p-value | 0.0094 | | 0.0786 | |
| Best overall response, n (%) |  |  |  |  |
| Stringent CR | 2 (4.2) | 1 (2.0) | 2 (10.0) | 1 (5.9) |
| ≥CR | 3 (6.3) | 3 (6.0) | 1 (5.0) | 1 (5.9) |
| VGPR | 15 (31.3) | 9 (18.0) | 5 (25.0) | 2 (11.8) |
| PR | 16 (33.3) | 13 (26.0) | 9 (45.0) | 7 (41.2) |
| MR | 8 (16.7) | 5 (10.0) | 2 (10.0) | 0 |
| SD | 4 (8.3) | 11 (22.0) | 1 (5.0) | 4 (23.5) |
| PD | 0 | 5 (10.0) | 0 | 0 |
| NE | 0 | 3 (6.0) | 0 | 2 (11.8) |
| Median DOR, months | 12.55 | 12.68 | 14.75 | 6.24 |
| HR (95% CI) | 0.95 (0.48-1.91) | | 0.73 (0.26-2.02) | |
| One-sided p-value | 0.4439 | | 0.2719 | |
| Median TTNT, months | 14.06 | 8.57 | 14.03 | 7.62 |
| HR (95% CI) | 0.57 (0.35–0.94) | | 0.54 (0.23–1.30) | |
| One-sided p-value | 0.0130 | | 0.0824 | |

*The following were considered high-risk cytogenetic abnormalities: del17p, t(4;14), t(14;16), or amplification of 1q21 (≥4 copies).
CI, confidence interval; CR, complete response; DOR, duration of response; HR, hazard ratio; MR, minimal response; NR, not reached; OR, odds ratio; ORR, overall response rate; OS, overall survival; PD, progressive disease; PFS, progression-free survival; PR, partial response; SD, stable disease; TTNT, time to next treatment; Vd, bortezomib and dexamethasone; VGPR, very good partial response; XVd, selinexor, bortezomib, and dexamethasone.

**Table S4. Efficacy outcomes by cytogenetic risk status, baseline disease characteristics, and treatment**

|  | **High-risk cytogenetics^*^** | | | | **Standard-risk cytogenetics** | | | |
| --- | --- | --- | --- | --- | --- | --- | --- | --- |
|  | **1 prior line** | | **2–3 prior lines** | | **1 prior line** | | **2–3 prior lines** | |
|  | **XVd**  **(n=35)** | **Vd**  **(n =32)** | **XVd**  **(n =35)** | **Vd**  **(n =39)** | **XVd**  **(n =64)** | **Vd**  **(n =67)** | **XVd**  **(n =61)** | **Vd**  **(n =69)** |
| Median PFS, months | 15.47 | 6.97 | 10.18 | 9.46 | 21.03 | 10.87 | 11.76 | 8.87 |
| HR (95% CI) | 0.67 (0.34–1.30) | | 0.82 (0.45–1.50) | | 0.59 (0.34–1.02) | | 0.62 (0.38–1.01) | |
| One-sided p-value | 0.1167 | | 0.2579 | | 0.0289 | | 0.0266 | |
| ORR, n (%) | 30 (85.7) | 20 (62.5) | 25 (71.4) | 21 (53.8) | 50 (78.1) | 45(67.2) | 44 (72.1) | 43 (62.3) |
| OR (95% CI) | 3.60 (1.10–11.80) | | 2.14 (0.82–5.63) | | 1.75 (0.80–3.82) | | 1.57 (0.75–3.29) | |
| One-sided p-value | 0.0152 | | 0.0610 | | 0.0809 | | 0.1186 | |
|  | **R-ISS Stage I–II** | | **R-ISS Stage III** | | **R-ISS Stage I–II** | | **R-ISS Stage III** | |
|  | **XVd**  **(n=63)** | **Vd**  **(n=61)** | **XVd**  **(n=6)** | **Vd**  **(n=8)** | **XVd**  **(n=110)** | **Vd**  **(n=116)** | **XVd**  **(n=6)** | **Vd**  **(n=8)** |
| Median PFS, months | 13.14 | 8.15 | 3.61 | 8.59 | 21.03 | 9.43 | 5.75 | 7.62 |
| HR (95% CI) | 0.64 (0.39–1.03) | | 1.53 (0.37–6.42) | | 0.52 (0.35–0.84) | | 1.15 (0.32–4.18) | |
| One-sided p-value | 0.0318 | | 0.7218 | | 0.0005 | | 0.5855 | |
| ORR, n (%) | 51 (81.0) | 34 (55.7) | 3 (50.0) | 5 (62.5) | 85 (77.3) | 74 (63.8) | 5 (83.3) | 5 (62.5) |
| OR (95% CI) | 3.38 (1.51–7.56) | | 0.60 (0.07–5.14) | | 1.93 (1.08–3.46) | | 3.00 (0.23–39.61) | |
| One-sided p-value | 0.0013 | | 0.6739 | | 0.0135 | | 0.2053 | |
|  | **Lenalidomide-treated** | | **Lenalidomide-naïve** | | **Lenalidomide-treated** | | **Lenalidomide-naïve** | |
|  | **XVd**  **(n=26)** | **Vd**  **(n=29)** | **XVd**  **(n=44)** | **Vd**  **(n=42)** | **XVd**  **(n=51)** | **Vd**  **(n=48)** | **XVd**  **(n=74)** | **Vd**  **(n=88)** |
| Median PFS, months | 12.22 | 5.75 | 12.91 | 9.46 | 8.54 | 7.62 | 21.03 | 10.61 |
| HR (95% CI) | 0.57 (0.28–1.15) | | 0.85 (0.47–1.53) | | 0.66 (0.38–1.13) | | 0.54 (0.33–0.89) | |
| One-sided p-value | 0.0568 | | 0.2933 | | 0.0624 | | 0.0075 | |
| ORR, n (%) | 16 (61.5) | 14 (48.3) | 39 (88.6) | 27 (64.3) | 36 (70.6) | 27 (56.3) | 58 (78.4) | 61 (69.3) |
| OR (95% CI) | 1.71 (0.59–5.02) | | 4.33 (1.41–13.35) | | 1.87 (0.81–4.28) | | 1.60 (0.78–3.28) | |
| One-sided p-value | 0.1642 | | 0.0039 | | 0.0702 | | 0.0973 | |
|  | **CrCl ≤60 mL/min** | | **CrCl >60 mL/min** | | **CrCl ≤60 mL/min** | | **CrCl >60 mL/min** | |
|  | **XVd**  **(n=21)** | **Vd**  **(n=23)** | **XVd**  **(n=49)** | **Vd**  **(n=48)** | **XVd**  **(n=35)** | **Vd**  **(n=47)** | **XVd**  **(n=90)** | **Vd**  **(n=89)** |
| Median PFS, months | 5.62 | 5.91 | 13.24 | 9.49 | 21.03 | 7.26 | 15.21 | 9.66 |
| HR (95% CI) | 0.96 (0.41–2.20) | | 0.62 (0.36–1.05) | | 0.35 (0.16–0.75) | | 0.74 (0.49–1.13) | |
| One-sided p-value | 0.4570 | | 0.0377 | | 0.0024 | | 0.0819 | |
| ORR, n (%) | 16 (76.2) | 12 (52.2) | 39 (79.6) | 29 (60.4) | 29 (82.9) | 28 (59.6) | 65 (72.2) | 60 (67.4) |
| OR (95% CI) | 2.93 (0.80–10.71) | | 2.56 (1.03–6.31) | | 3.28 (1.14–9.42) | | 1.26 (0.66–2.38) | |
| One-sided p-value | 0.0510 | | 0.0201 | | 0.0122 | | 0.2424 | |

*Patients were considered high-risk if they presented with ≥1 of the following cytogenetic abnormalities: del17p, t(4;14), t(14;16), or amplification of 1q21 (≥4 copies).
CI, confidence interval; CrCl, creatinine clearance; HR, hazard ratio; OR, odds ratio; ORR, overall response rate; PFS, progression-free survival; R-ISS, Revised International Staging System; Vd, bortezomib and dexamethasone; XVd, selinexor, bortezomib, and dexamethasone.
